# Renal DCE-MRI Model Selection Using Bayesian Probability Theory

**DOI:** 10.18383/j.tom.2015.00133

**Published:** 2015-09

**Authors:** Scott C. Beeman, Patrick Osei-Owusu, Chong Duan, John Engelbach, G. Larry Bretthorst, Joseph J. H. Ackerman, Kendall J. Blumer, Joel R. Garbow

**Affiliations:** 1 Departments of Radiology,; 2 Cell Biology and Physiology,; 3 Chemistry, and; 4 Medicine, Washington University, St. Louis, MO

**Keywords:** Bayesian model selection, dynamic contrast enhanced MRI, signal modeling

## Abstract

The goal of this work was to demonstrate the utility of Bayesian probability theory-based model selection for choosing the optimal mathematical model from among 4 competing models of renal dynamic contrast-enhanced magnetic resonance imaging (DCE-MRI) data. DCE-MRI data were collected on 21 mice with high (n = 7), low (n = 7), or normal (n = 7) renal blood flow (RBF). Model parameters and posterior probabilities of 4 renal DCE-MRI models were estimated using Bayesian-based methods. Models investigated included (1) an empirical model that contained a monoexponential decay (washout) term and a constant offset, (2) an empirical model with a biexponential decay term (empirical/biexponential model), (3) the Patlak–Rutland model, and (4) the 2-compartment kidney model. Joint Bayesian model selection/parameter estimation demonstrated that the empirical/biexponential model was strongly favored for all 3 cohorts, the modeled DCE signals that characterized each of the 3 cohorts were distinctly different, and individual empirical/biexponential model parameter values clearly distinguished cohorts of low and high RBF from one another. The Bayesian methods can be readily extended to a variety of model analyses, making it a versatile and valuable tool for model selection and parameter estimation.

## Introduction

Renal dynamic contrast-enhanced magnetic resonance imaging (DCE-MRI) is a powerful technique that can noninvasively quantify and map empirical and physiological parameters that provide information on renal function. For example, DCE-MRI can quantify and map renal blood flow (RBF) and the glomerular filtration rate (GFR) (1–6), important clinical determinants of renal function that are otherwise traditionally measured based on filtering para-aminohippuric acid and inulin into the urine (measures that report on the combined RBF and GFR of both kidneys). By mapping function parameters, renal DCE-MRI can potentially yield information on spatially heterogeneous renal diseases such as focal and segmental glomerular sclerosis and thus may be preferable to traditional plasma- and urine-based measures. When applied in humans, renal DCE-MRI can potentially provide noninvasive, quantitative insight into a patient's renal health, as well as inform on basic human renal physiology. In rodents, renal DCE-MRI permits quantitative, serial measures of kidney structure and function in support of drug discovery (nephrotoxicity) and characterization of renal function in genetically manipulated animals.

Renal DCE-MRI involves serial imaging of the kidney using a T1-weighted MRI sequence to observe the passage of a bolus of gadolinium-containing contrast agent (CA) through the kidney. From these data, dynamic parameters can be quantified by fitting descriptive mathematical models—those that provide an approximate representation of a complex system—to the MRI data. Renal DCE-MRI mathematical models can loosely be categorized as either pharmacokinetic compartmental or empirical models. Pharmacokinetic compartmental models are derived based on approximations of the known physiological processes that underlie the MRI signal. Many of these models aim to measure fundamental physiological parameters such as RBF and GFR (1–6). Pharmacokinetic models typically require knowledge of the arterial input function (AIF), which describes the passage of the CA bolus through the vasculature. The input function is often measured distal from the tissue of interest and thus may not accurately describe bolus dynamics in voxels of interest. Furthermore, errors in AIF measurements (delay of bolus arrival, dispersion of bolus in tissues, partial volume effects, flow artifacts) can markedly influence model parameter estimates (7). AIF measurements are particularly difficult in small-animal models of disease. As a consequence, it can be challenging to make accurate physiological measurements with pharmacokinetic models. Alternatively, empirical models can be used to characterize DCE-MRI data using simple, logically chosen mathematical functions that may qualitatively reflect underlying physiology. Although empirical models typically do not require a direct theoretical connection to the underlying physiology, appropriately selecting mathematical functions can yield useful descriptions of that physiology.

Ideally, a library of models would be compared against one another to determine which model best represents a dataset. Selecting the “best” mathematical model for a dataset requires accounting not only for goodness of fit, reflected in the model versus data residuals, but also the complexity of the model (8–10), that is, the number of variable parameters in the model. This idea is generally expressed as a statement of Occam's razor or the law of parsimony—that all other things being equal, a simpler model, with fewer parameters, is favored over a more complex, highly parameterized model. However, more complex models will often better fit the experimental data. To avoid overparameterization, a best-fit model must balance goodness of fit versus complexity.

Cox's theorem (11) and its further elaboration by Jaynes (12–14) states that Bayesian probability theory is the optimal method for making inferences about data (ie, it optimally balances goodness of fit vs complexity). Bayesian probability theory answers the following question: given the data, and all prior information, what is the probability that a given hypothesis is true? Bayesian probability theory incorporates prior knowledge (in the form of prior probabilities) that informs the calculation and effectively provides a goodness-of-fit penalty for each parameter added to a data model. Based upon the data and all prior information, Bayesian calculations generate marginalized posterior probability distributions. These probability distributions provide information regarding model selection and estimated parameter values, including provision of the uncertainties associated with the derived results (quantified by the widths of the probability distributions). Thus, Bayesian probability theory can provide optimal estimates of the parameters that characterize a model and a probabilistic ranking of members of a library of competing data models.

We apply Bayesian probability theory jointly on cohort datasets herein to select the optimal (most probable) model among 4 renal DCE-MRI models. We then apply this methodology to differentiate 3 cohorts of mice with different blood flow rates.

## Methods

### Animal Preparation and Imaging

All animal experiments were approved by the Washington University Division of Comparative Medicine. This study aimed to modulate renal dynamics by modulating RBF using a nitric oxide synthase inhibitor, *N*-nitro-l-arginine methyl ester (l-NAME), to reduce RBF and an angiotensin receptor antagonist, losartan, to increase RBF. To confirm the effects of l-NAME and losartan on RBF before DCE-MRI experiments, mice were anesthetized with 1.5% isoflurane, and their left jugular vein and right carotid arteries were catheterized for drug delivery and arterial blood pressure measurement, respectively. The left kidney was accessed via the retroperitoneal cavity. Kidney regional blood flow was continuously monitored by means of 2 laser Doppler flow probes (Advanced Laser Doppler Flowmeter 21, ADVANCE, Tokyo, Japan), 1 placed on the surface of the left pole and the other, the size of a 23-gauge needle, inserted 7 mm into the kidney to measure renal cortical and medullary blood fluxes (CBFs and MBFs), respectively. Mice were stabilized under anesthesia for 15 minutes after surgical procedures. One group of mice (n = 3) received 30 mg/kg l-NAME intravenously, a second group of mice (n = 3) received 20 mg/kg losartan intravenously, and a third group (n = 3) served as the control. Mean arterial blood pressure (MAP), CBF, and MBF were continuously monitored before and after each injection. At the end of the experiment, the kidney was fixed in 10% formalin, and medullary probe placement was verified by visually inspecting the probe track under light microscopy. The acute effects of l-NAME and losartan on blood pressure and kidney regional blood flow were determined by computing the percentage change relative to baseline values.

Renal DCE-MRI experiments were performed on a small-animal scanner equipped with an Agilent DirectDrive console (Agilent Technologies, Santa Clara, CA) and built around a 4.7-T horizontal bore magnet (Oxford Instruments, Abingdon, UK). Mice were anesthetized with 1% isoflurane, and their left jugular veins were catheterized. To vary RBF, mice received 30 mg/kg l-NAME (n = 7) intravenously to reduce RBF, 20 mg/kg losartan (n = 7) intravenously to increase RBF, or remained naive as controls (n = 7). Mice were imaged supine using a laboratory-built, actively decoupled volume transmitting/surface receiving coil pair, with the receiving coil placed directly under the left kidney. DCE-MRI data were collected using a gradient-recalled echo (GRE) pulse sequence with the following parameters: echo time, 2.7 milliseconds; repetition time, 30 milliseconds; flip angle, 30°; matrix, 64 × 64; slice thickness, 1 mm; field of vision, 30 mm^2^; temporal resolution, 5.12 seconds; and total scan time, 17 minutes and 3 seconds. At 123 seconds after the start of the DCE-MRI series (frame 24 of the time series), a bolus of 100 μL of 16 mM gadobenate dimeglumine contrast agent (MultiHance, Bracco Imaging, Monroe Township, NJ) was administered for 3 seconds via a jugular vein catheter using a syringe pump (Harvard Clinical Technology, Natick, MA). Care was taken to eliminate saline from the catheter line before the DCE-MRI experiments to ensure that the entire 100 μL of contrast agent was administered. To calculate the baseline longitudinal relaxation rate constant (*R*_1_) of the kidney cortex, 8 GRE images with flip angles of 2, 5, 7, 9, 15, 20, 25, and 35° (variable flip-angle experiment) were collected before the DCE-MRI experiment (15).

### Data Analysis

Regions of interest (ROIs) outlining only the renal cortex were manually defined from high-resolution, T2*-weighted GRE images of the kidney. Raw arterial input functions for each mouse were defined as the mean measured signal in the renal artery. DCE-MRI signal versus time datasets were converted to apparent CA concentration using the baseline *R*_1_ estimates and standard procedures (1). Bayesian probability theory-based methods, implemented using a Markov-chain Monte Carlo simulation (details below in “Bayesian-Based Model Selection”), were applied on both an ROI- and voxel-wise basis to estimate both model probability and model parameter values. The posterior probabilities of each model and its parameters were computed using laboratory-developed software (further algorithm details and software are available for free download at http://bayesiananalysis.wustl.edu). Four competing signal models were evaluated on both an ROI and voxel-wise basis: (1) an empirical model containing a monoexponential signal decay (washout) term and a constant offset (empirical/monoexponential + C model), (2) an empirical model with a biexponential signal decay term (empirical/biexponential model), (3) the Patlak– Rutland model (2–4, 16), and (4) the 2-compartment kidney model (4, 16). All models are detailed in the sections that follow.

Joint Bayesian analysis, in which model selection and parameter estimates are calculated jointly for the entirety of each cohort (ie, a single posterior probability distribution function [PDF] is produced for each parameter in each cohort), was performed on ROI data. As will be described, the empirical/biexponential model was heavily favored for all 3 cohorts. To determine whether Bayesian analysis of the kidney DCE-MRI data could distinguish the 3 cohorts of animals (normal, high, and low RBF), a series of empirical/biexponential models was devised to determine whether the 3 cohorts were the same, different, or both. In practical terms, Bayesian model selection made these determinations by comparing 1 empirical/biexponential model in which all cohorts shared the same model parameters, 1 empirical/biexponential model in which all cohorts had unique model parameters, and 3 empirical/biexponential models in which 2 cohorts shared the same parameters and the other cohort had unique parameters.

Having established that the 3 cohorts were different from one another (vide infra, see “Results: ROI Parameter Estimation and Model Selection”), the PDFs for each empirical/biexponential model parameter were compared across cohorts to determine which parameters were most responsible for the observed difference between cohorts. Differences between PDFs were calculated, and parameters were considered different between cohorts if the 95% confidence interval of the difference in the PDFs did not overlap with 0 (17). Unless otherwise noted, data are presented as mean ± SD.

### Pharmacokinetic (Physiologic) Models

Two pharmacokinetic models were considered: the Patlak–Rutland model (2–4, 16) and the 2-compartment kidney model. The Patlak–Rutland model is given by

(1) $$K\left(t\right)=\upsilon_{p}A_{p}\left(t\right)+\frac{GFR}{V}\int_{0}^{t}A_{p}\left(u\right)du,$$

where *K*(*t*) is the MRI signal amplitude, *v*_*p*_ is the apparent vascular volume fraction, *A_p_*(*t*) is the apparent CA plasma concentration, *t* is time (measured from the start of the MRI scan), GFR is the glomerular filtration rate, and *V* is the renal cortical tissue volume. The 2-compartment kidney model is given by

(2) $$K\left(t\right)=\upsilon_{p}A_{p}\left(t\right)+\frac{\text{GFR}}{V}\int_{0}^{t}A_{p}\left(u\right)\exp\left(-k_{\text{out}}\left(t-u\right)\right)du,$$

where *k*_out_ is the rate constant that governs the outflow of CA from tubules.

### Empirical Models

Both empirical models take the following general form:

(3) K(t)=f(t)×g(t),

in which *f*(*t*) describes the CA wash-in and *g*(*t*) the CA washout; *f*(*t*) is modeled with the following cumulative log-logistic equation:

(4) f(t)=A1+(tB)−C,

where *A* is the amplitude of the CA wash-in (interpreted as the peak CA concentration), *t* is time (measured from the start of the MRI scan), *B* is the inflection time point of the CA wash-in (the time point of the maximum rate of CA accumulation), and *C* is the rate of CA wash-in (the slope) near *t* = *B*; *g*(*t*), the CA washout, is modeled as either a monoexponential decay to a constant offset or a biexponential decay. The former model is given by

(5) $$g_{mono}\left(t\right)=\exp\left(-\text{α}\left(t-\text{τ}\right)\right)+c,$$

where *a* is the decay rate constant (which informs the combined clearance of CA from the renal cortex via filtration into the tubules and washout to the venous system), τ is the time at which the CA bolus was injected, and *C* is the constant offset. The biexponential decay model is given by

(6) $$g_{bi}\left(t\right)=F_{f}\exp\left(-\alpha_{f}\left(t-\tau \right)\right)+F_{s}\exp\left(-{\text{α}}_{s}\left(t-\text{τ}\right)\right),$$

in which *F*_*f*_ and *F*_*s*_ are the fractional amplitudes of the fast and slow exponential decay terms, respectively, and α_*f*_ and α_*s*_ are the corresponding decay-rate constants (which inform the clearance of CA from the renal cortex via washout to the venous system and filtration into the tubules, respectively). The fractional amplitudes are constrained by *F*_*f*_ + *F*_*s*_ = 1; thus, either fractional amplitude can be reported equivalently—the fractional amplitude of the slow component (filtration) is reported herein.

A monoexponential decay without a constant offset and a biexponential decay that included one were also considered. However, a Bayesian assessment revealed both of these models to be highly improbable; for this reason, these models have been excluded from further discussion.

### Bayesian-Based Model Selection

The posterior probability for each of the models previously defined was calculated using Bayes' theorem (14):

(7) $$P\left(M|DI\right)=\frac{P\left(M|I\right)P\left(D|MI\right)}{P\left(D|I\right)},$$

where P(M|DI) is the posterior probability for a model *M* given the data *D* and the prior information *I*; P(M|I) is the prior probability for the model given the prior information; P(D|MI) is the marginal direct probability for the data given the model and prior information; and P(D|I) is the direct probability for the data given the prior information. To calculate the posterior probability of the model given the data and prior information, one must calculate the direct probability for the data given the model and the prior information. For example, in the case of the Patlak–Rutland pharmacokinetic model and its 2 calculated kinetic parameters GFR and *v*_*p*_, the expansion of P(D|MI) takes the following form:

(8) $$P\left(D|MI\right)=\int dGFRd\upsilon_{p}P\left(GFR\upsilon_{p}|MI\right)P\left(D|GFR\upsilon_{p}MI\right),$$

where $P\left(GFR\upsilon_{p}|MI\right)$ is the joint prior probability for GFR and *v*_*p*_ given the model and the prior information and $P\left(D|GFR\upsilon_{p}MI\right)$ is the direct probability for the data given the parameters (GFR and *v*_*p*_), the model, and the prior information. It is critical to note here that Bayesian probability theory demands accounting for the entire hypervolume defined by the range of parameter values. Each hypervolume defined by the parameters contributes directly to the posterior probability of the model given the data and prior information. However, each hypervolume defined by the prior probability for the parameter is weighted by the likelihood of the data given those parameters. The posterior probabilities for the parameters and each model defined previously were calculated using custom-written Bayesian analysis software that employed a Markov-chain Monte Carlo simulation, in which the model is merely considered another discrete parameter to be sampled.

In the Markov-chain Monte Carlo simulation, 50 simulations were run simultaneously in parallel with thermodynamic integration to sample the posterior probability for the parameters and the model. Thermodynamic integration slowly brought the 50 simulations to a static equilibrium state. Once the system was in equilibrium, 50 samples of each of the 50 simulations were gathered, so 2,500 total parameter samples were used to characterize the density distribution of the posterior probabilities for the parameters and the models.

All calculations were performed on a Dell Power Edge R900 with 4 6-core (24 central processing units) 2.4 GHz Xeon processors with 48 gigabytes of memory. Model selection and parameter estimation on ROI data took 45 to 75 seconds for each cohort. Voxel-wise model selection and parameter estimation took 12 hours for each dataset.

## Results

### Effects of l-NAME and Losartan on Blood Pressure and Regional Blood Flow in the Kidney

**Figure 1.**
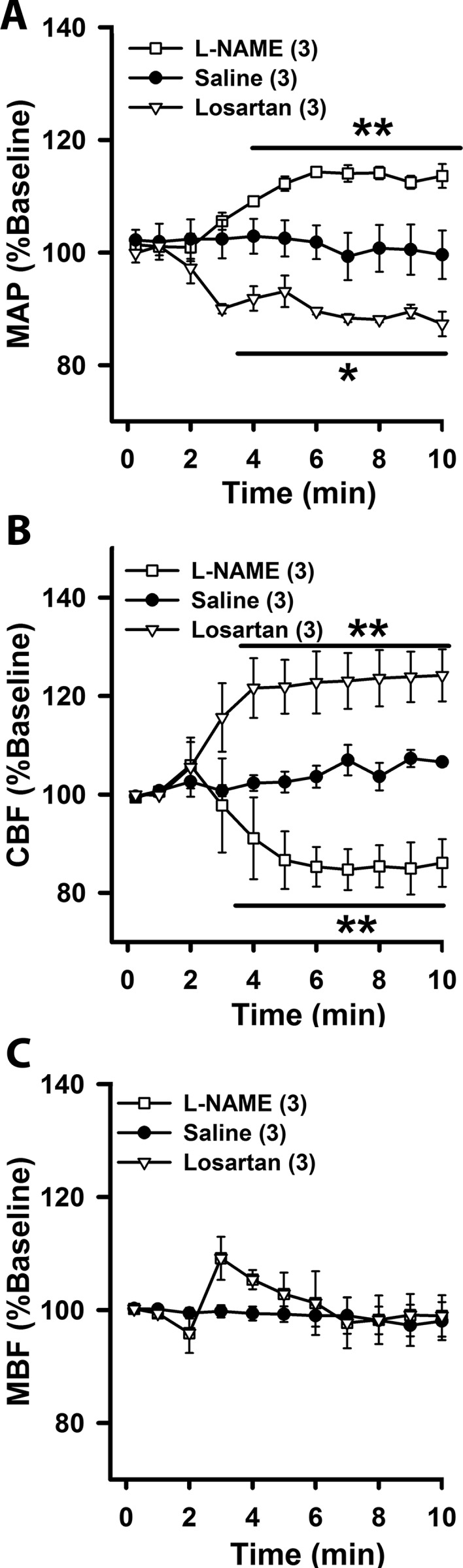
The effects of acute intravenous administration of 10 L of saline (n = 3), 30 mg/kg l-NAME (n = 3), and 20 mg/kg losartan (n = 3) on MAP (A), renal CBF (B), and renal MBF (C). Data are expressed as percentage changes from baseline values determined as the mean values over 2 minutes before drug or vehicle administration. All values are mean ± SEM. *P < .05 and **P < .01 versus saline.

The effects of l-NAME and losartan were confirmed using 2 laser Doppler flow probes before the renal DCE-MRI experiments. l-NAME administration increased MAP (12% above baseline; Figure 1A) and decreased CBF (15% relative to baseline; Figure 1B). Conversely, losartan administration decreased MAP (10% below baseline; Figure 1A) and increased CBF (23% above baseline; Figure 1B). The effects of l-NAME and losartan on CBF were sustained (>9 minutes; Figure 1B), whereas their effects on MBF were transient (<4 minutes; Figure 1C).

### ROI Parameter Estimation and Model Selection

ROIs and AIFs were manually defined for each of the 21 DCE-MRI datasets.

#### Joint (Cohort) Model Selection and Parameter Estimation

**Figure 2.**
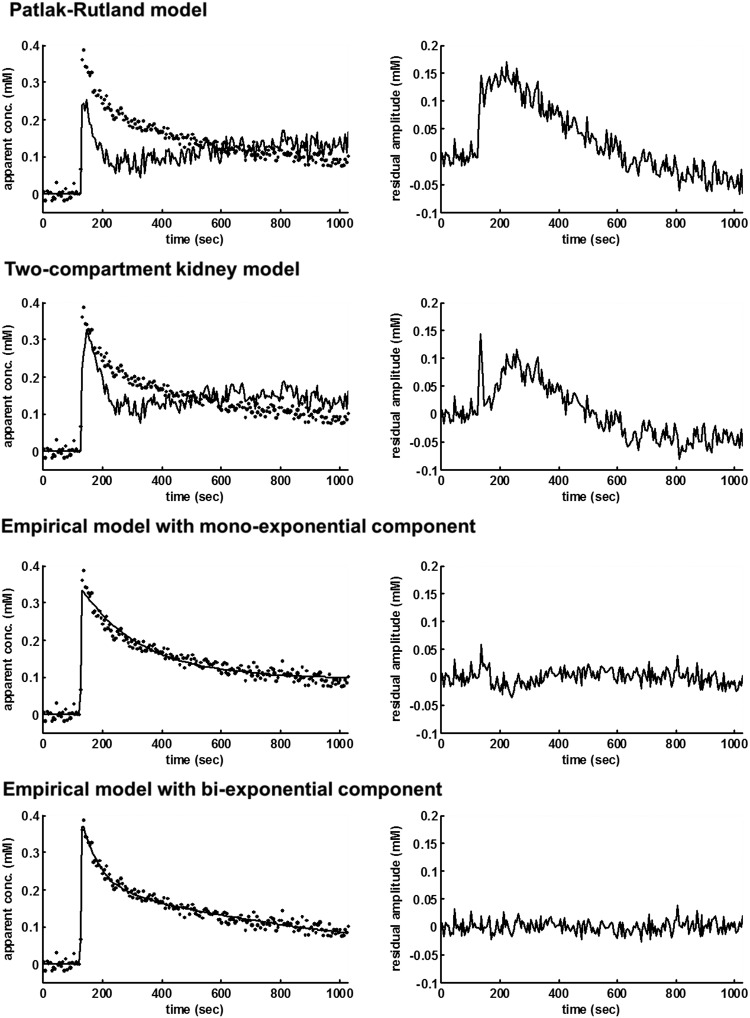
Representative DCE-MRI data from a single animal, together with the Bayesian modeling of these data using 2 pharmacokinetic and 2 empirical models (left), and residuals (right). The empirical/biexponential model was calculated to be the most probable signal model from among the 4 compared models for each of the animal cohorts.

**Figure 3.**
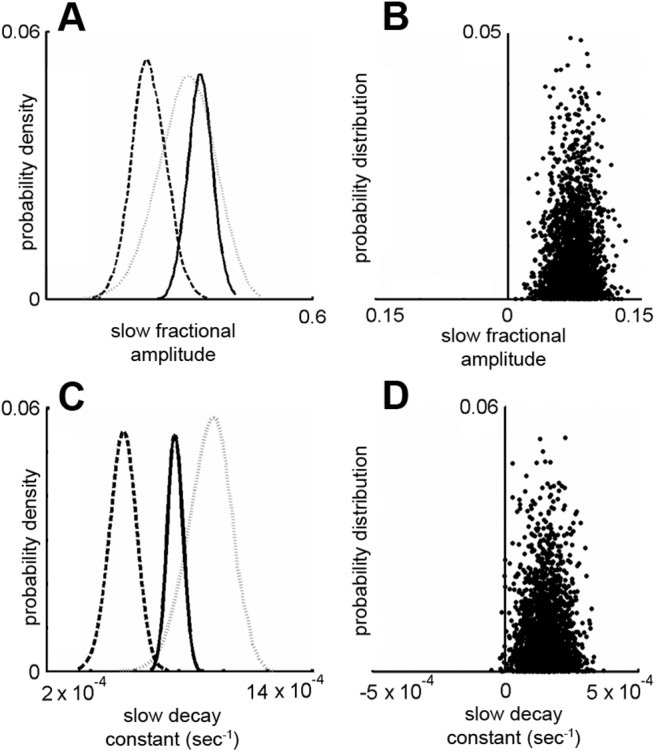
(A, C) Bayesian-estimated posterior probability densities of the empirical/biexponential model's joint fractional amplitudes of the washout and joint slow decay-rate constants, respectively, for each cohort. The losartan-treated (high RBF) group is represented by the solid black line, the l-NAME (low RBF) group by the dashed black line, and the control group by the dotted gray line. (B, D) The difference in the posterior probability distributions for the joint fractional amplitudes of the washout and joint slow decay-rate constants, respectively, calculated on the high and low RBF cohorts. From among the empirical/biexponential model joint parameter estimates, the fractional amplitudes of the washout terms (A) and the slow decay-rate constants (C) differed between mouse cohorts of high and low RBF; that is, the 95% confidence interval of the difference in the probability distributions did not overlap with 0 (B and D).

Intracohort datasets were analyzed jointly (7 datasets per cohort) using Bayesian probability theory-based methods to (1) identify the most probable signal model from among the 4 competing models considered, (2) discern cohorts of differing RBF, and (3) compute optimized model parameters for the most probable model per cohort (Figure 2). Joint Bayesian model selection calculated the empirical/biexponential model to be >847 e-folds or, equivalently, exp(847) times more probable than the other models for all cohorts combined. Intracohort joint Bayesian model selection chose the empirical/biexponential model as the favored model for each cohort individually (>147, 208, and 449 e-folds more probable than the other models for the control, low RBF, and high RBF cohorts, respectively). Based upon an evaluation of empirical/biexponential models in which all cohorts were considered all the same, all different, or pairwise the same (with the third cohort being different), Bayesian model selection assigns an overwhelming probability to the hypothesis that the 3 cohorts are all different (>8 e-folds or, equivalently, 3,000 times more probable than all others). From among the empirical/biexponential model parameters (Table 1), the slow decay-rate constants and the fractional amplitudes of the washout terms differed individually between mouse cohorts of high and low RBF (ie, the 95% confidence interval of the difference in the probability distributions did not overlap with 0; Figure 3B, D). The fast decay-rate constants discerned the control group from the high and low RBF groups.

**Table 1.** Joint Parameter Values Estimated Using the Empirical/Biexponential Model

	Joint Empirical/Biexponential Model Parameter Values					
	CA amplitude (mM)	Inflection Point (s)	Slope (×10^1^ mM/s)	Slow Decay-Rate Constant (×10^−4^ s^−1^)	Fast Decay-Rate Constant (×10^−2^ s^−1^)	Slow Fractional Amplitude (AU)
Control	0.5 ± 0.2	7.0 ± 0.3	8 ± 1	9.4 ± 1.0	1.0 ± 0.1	0.48 ± 0.03
Low flow	0.5 ± 0.2	7.4 ± 0.2	10 ± 3	6.6 ± 0.5	1.6 ± 0.1	0.42 ± 0.01
High flow	0.5 ± 0.2	6.9 ± 0.2	9 ± 1	8.1 ± 0.3	1.6 ± 0.1	0.49 ± 0.01

#### Voxel-Wise Analyses

**Figure 4.**
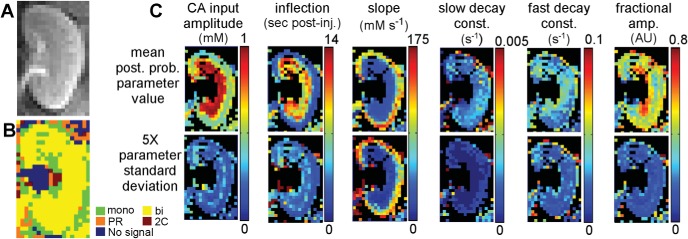
(A) Representative gradient-recalled echo image from a DCE-MRI image series. (B) Representative voxel-wise results of Bayesian model selection, where green, yellow, orange, red, and blue indicate Bayesian probability theory-preference of the empirical/monoexponential + C model, the empirical/biexponential model, the Patlak–Rutland model, the 2-compartment kidney model, and no signal, respectively. (C) Maps of derived empirical/biexponential model parameters (top) and their standard deviations (amplified 5×; bottom).

When the datasets were analyzed on a voxel-wise basis, the empirical/biexponential model was the preferred model for the vast majority of voxels within the renal cortex and medulla (Figure 4B, yellow). The empirical/monoexponential + C model (green) is preferred in the spleen, shown on the right edge of these images, and the outermost regions of the renal pelvis. In this representative dataset, neither the Patlak–Rutland nor the 2-compartment kidney models were associated with a particular tissue type, and these models accounted for a small minority of voxels in the analyses. The analysis excluded the renal pelvis, where the CA accumulated at high concentrations and thus induced a significant T2* effect that was not included in the models employed in this study. Empirical/biexponential model parameters and their standard deviations were mapped (Figure 4C). Interestingly, these parameter maps highlight a number of anatomic features of the kidney. The CA input amplitude and inflection time point are highest in the tubule-bearing medulla of the kidney, where renal dynamics are mostly diffusion-limited and one would expect the CA to slowly accumulate to high concentrations. The slope of the wash-in is maximized in the cortex, where dynamics are mostly driven by intracapillary flow and one would expect both the wash-in and washout of the CA to be rapid.

## Discussion

In this proof-of-principle study, Bayesian probability theory-based methods are shown to be powerful tools for selecting the most probable kinetic model for DCE-MRI datasets and for estimating DCE-MRI model parameters. Joint (cohort) model selection and parameter estimation revealed the empirical/biexponential model to be the most probable signal model for all 3 cohorts (normal, high, and low RBF) of mice. Bayesian model selection broadly answered whether these cohorts were the same or different without needing to compare specific parameter estimates. The 3 cohorts of mice of varying RBF were readily discerned in this manner. From among the empirical/biexponential model parameters, the slow decay-rate constant and the fractional amplitudes of the washout terms were different between cohorts of mice with high and low RBF (Figure 3).

Renal DCE-MRI is particularly difficult to perform in rodents. Many of the challenges associated with DCE-MRI in rodents stem from the small and often difficult-to-find feeding arteries from which an AIF can be sampled. Although care was taken to capture the renal artery in the renal DCE-MRI datasets, these same challenges affected the results of this study, and it is likely that the empirical models were favored over the pharmacokinetic models largely because of poor AIF data in these mouse kidney images. Alternatively, pharmacokinetic models may be favored in human studies in which large feeding vessels are more readily sampled for AIFs. From this work, we conclude that Bayesian probability theory is a powerful tool for assessing the fidelity of kinetic models given DCE-MRI data and that, in general, flexible empirical models may provide more robust dynamic parameter estimates in preclinical DCE-MRI studies in which quality AIFs are difficult to reliably sample (at the expense of a direct report on typical physiological parameters).

Cortical ROI datasets were used in much of this study to determine the most probable kinetic model and to discern treated animal groups. However, Bayesian model selection and parameter estimation can also be applied on a voxel-wise basis. In this study, we found that the vast majority of cortical and medullary voxels were best fit by the empirical/biexponential model (Figure 4B). Still, a small portion of the spleen was captured in these images and, in the spleen, the empirical/monoexponential + C model was favored. Again, we speculate that the absence of Bayes-favored pharmacokinetic models in these voxel-wise analyses resulted from poorly resolved/sampled AIFs. Bayesian model selection, when applied on a voxel-wise basis, may thus be useful in mapping the distribution of favored models—a factor that may supplement model parameter estimates by yielding information about tissue microstructure and function.

Although correlating empirical model parameters with physiology was not an explicit goal of this work, it is interesting to note how the voxel-wise analyses hint at the physiological relevance of the empirical/biexponential model (Figure 4). The CA wash-in amplitude parameter, interpreted as the peak CA concentration, suggests that the renal medulla will experience a higher CA concentration than the cortex. This makes physiological sense because the CA transits quickly across the glomerular filtration barrier (in the cortex) into the tubules (mostly in the medulla), through which the agent traverses slowly, thus allowing buildup of CA. The CA wash-in inflection parameter, interpreted as the time point at which the rate of CA accumulation is greatest, suggests that the renal cortex and peritubular capillaries accumulate CA first, followed by the medulla (tubules). This too makes physiological sense because the agent originates in the glomerular intracapillary space in the cortex and traverses the capillary wall into the tubule in the medulla. Finally, the CA wash-in slope parameter, interpreted as the peak rate of CA accumulation, is highest in the renal cortex. This again is physiologically reasonable because the cortex is where the CA quickly filters through the glomerular slit diaphragm into the tubule lumen. The high rate of CA delivery from the vasculature into tubules, driven by renal perfusion pressure, causes a sharp increase in CA concentration (large slope parameter) over a very short period of time. Note that the CA wash-in slope represents an instantaneous maximum rate of accumulation; thus, its large value is offset by the very short time period over which CA accumulates at this rate. The empirical parameters that govern the wash-out of the CA combine to account for the clearance of CA from the cortex via washout through the venous system or filtration through the glomerular capillary wall and tubules into the urinary space.

This study was not an exhaustive assessment of all possible/reported pharmacokinetic and empirical models. In addition, we do not necessarily advocate the use of empirical models over pharmacokinetic models in all circumstances. Still, it is important to note that in this work Bayesian probability theory heavily favored the use of empirical models and that the empirical/biexponential model was the only model that could discern cohorts with high blood flow from those with low blood flow. We speculated previously that the empirical models were favored in this study because quality AIFs are difficult to obtain in preclinical models. Similar methods may indeed lead to a preference of pharmacokinetic models in humans, wherein AIFs are more easily sampled and of higher fidelity. Furthermore, the empirical models used in this study were hierarchical and shared a common wash-in function. In principle, the wash-in parameters for cohort studies could be analyzed jointly across the hierarchical empirical models regardless of whether monoexponential or biexponential decay functions were favored. The joint analysis of common empirical terms could improve the power and fidelity of intergroup analyses in which mono- and biexponential wash-out terms are favored. Exploring the hierarchical nature of these empirical models will be the focus of future efforts.

This study is presented as a proof of principle that Bayesian probability theory—the optimal method for making inferences about data (11–14)—can be used to compare multiple renal DCE-MRI models, select the most probable signal model, broadly discern test cohorts, and optimally estimate parameter values. This work also shows that empirical DCE-MRI models, which do not require difficult-to-sample AIFs, may be more suitable than pharmacokinetic models in a preclinical setting. This methodology can be readily extended to a wide variety of analyses, making it a versatile and valuable tool for model selection and parameter estimation.
